# Cyclooxygenase-2 Induction by Arsenite through the IKKβ/NFκB Pathway Exerts an Antiapoptotic Effect in Mouse Epidermal Cl41 cells

**DOI:** 10.1289/ehp.9588

**Published:** 2006-12-14

**Authors:** Weiming Ouyang, Dongyun Zhang, Qian Ma, Jingxia Li, Chuanshu Huang

**Affiliations:** Nelson Institute of Environmental Medicine, New York University School of Medicine, Tuxedo, New York, USA

**Keywords:** apoptosis, arsenite, carcinogenesis, COX-2, NFκB

## Abstract

**Background:**

Arsenic contamination has become a major public health concern worldwide. Epidemiologic data show that long-term arsenic exposure results in the risk of skin cancer. However, the mechanisms underlying carcinogenic effects of arsenite on skin remain to be studied.

**Objectives:**

In the present study we evaluated cyclooxygenase-2 (COX-2) expression, the signaling pathways leading to COX-2 induction, and its antiapoptotic function in the response to arsenite exposure in mouse epidermal JB6 Cl41 cells.

**Methods:**

We used the luciferase reporter assay and Western blots to determine COX-2 induction by arsenite. We utilized dominant negative mutant, genetic knockout, gene knockdown, and gene overexpression approaches to elucidate the signaling pathway involved in COX-2 induction and its protective effect on cell apoptosis.

**Results:**

The induction of COX-2 by arsenite was inhibited in Cl41 cells transfected with IKKβ-KM, a dominant mutant inhibitor of kβ (Ikβ) kinase (IKKβ), and in IKKβ-knockout (IKKβ^−/−^) mouse embryonic fibroblasts (MEFs). IKKβ/nuclear factor κB (NFκB) pathway-mediated COX-2 induction exerted an antiapoptotic effect on the cells exposed to arsenite because cell apoptosis was significantly enhanced in the Cl41 cells transfected with IKKβ-KM or COX-2 small interference RNA (siCOX-2). In addition, IKKβ^−/−^ MEFs stably transfected with COX-2 showed more resistance to arsenite-induced apoptosis compared with the same control vector–transfected cells.

**Conclusions:**

These results demonstrate that arsenite exposure can induce COX-2 expression through the IKKβ/NFκB pathway, which thereby exerts an antiapoptotic effect in response to arsenite. In light of the importance of apoptosis evasion during carcinogenesis, we anticipate that COX-2 induction may be at least partially responsible for the carcinogenic effect of arsenite on skin.

Arsenic contamination has become a major public health concern worldwide, especially in Asia. Epidemiologic data show that long-term arsenic exposure results in the risk of various cancers [Bettley and O’Shea 1975; International Agency for Research on Cancer (IARC) 1980; Landolph 1994; Nriagu 2002], especially in the lung and skin via inhalation and ingestion (Landolph 1994). High arsenic concentrations in drinking water (0.35–1.14 mg/L) caused increased risks of cancer of the skin, bladder, kidney, lung, and colon (National Research Council 1999). The skin cancers associated with arsenite exposure include Bowen’s disease (carcinoma *in situ*), basal cell carcinoma, and squamous cell carcinoma (Tseng et al. 1968; Yu et al. 2006).

The mouse skin model of multistage carcinogenesis has demonstrated that cancer development results from the coordination of genetic mutation and alterations of epigenetic factors, including the machineries regulating cell proliferation and apoptosis (Hecker 1987; Zoumpourlis et al. 2003). Acquiring the capacity to evade apoptosis is a hallmark of most cancers (Hanahan and Weinberg 2000). Under normal circumstances, DNA-damaged or mutated cells are eliminated by apoptosis. Acquired resistance to apoptosis is a critical molecular event during carcinogenesis, and disruption of apoptosis has been shown to play a major role in tumor formation and malignant progression (Hanahan and Weinberg 2000; Hickman 2002). Whereas the induction of cell proliferation by arsenite has been extensively studied, the events implicated in regulating the apoptosis of skin cells exposed to arsenite remain largely unknown.

Cyclooxygenase (COX), the rate-limiting enzyme in the conversion of arachidonic acid to prostanoids (Sheng et al. 2001; Smith et al. 1996), exists as two distinct isoforms (Feng et al. 1993). COX-2 is an inducible immediate-early gene. Its expression is low or non-detectable in most tissues, but it can be readily induced in response to cell activation by cytokines, growth factors, and tumor promoters (Feng et al. 1993; Smith et al. 1996). Increasing evidence indicates that COX-2 is related to skin cancer development. Mice deficient in COX-2 develop 75% fewer tumors than their wild type littermates when subjected to a 9,10-dimethylbenz[*a*]anthracene/12-*O*-tetradecanoylphorbol-13-acetate two-stage chemical carcinogenesis protocol (Tiano et al. 2002). Moreover, oral administration of specific COX-2 inhibitors is effective in reducing ultraviolet-B–induced skin carcinogenesis by up to 90% (Fischer et al. 1999). Although the exact mechanisms remain to be extensively investigated, COX-2 is thought to contribute to carcinogenesis mainly by promoting cell proliferation and antagonizing cell apoptosis (Krysan et al. 2005; Tsujii and DuBois 1995; Wang et al. 2005).

The role of COX-2 in apoptosis resistance and carcinogenesis suggests that COX-2 may be involved in the regulation of apoptosis of skin cells exposed to arsenite. Therefore, in the present study we examined the effect of arsenite exposure on COX-2 expression in mouse epidermal JB6 Cl41 cells, and we further investigated the role of COX-2 in apoptosis resistance during arsenite exposure. The results showed that exposure to arsenite caused significant COX-2 expression through the inhibitor of κβ (Iκβ) kinase (IKKβ)/nuclear factor κB (NFκB) pathway, which thereby played an important role in antagonizing the apoptosis induced by arsenite. These results suggest that COX-2 induction in arsenite-exposed skin cells may facilitate skin cancer development by conferring an apoptosis resistance and supporting the survival of the cells with genetic alterations that are usually eliminated by apoptosis.

## Materials and Methods

### Cell culture

Mouse epidermal JB6 Cl41 cells and their stable transfectants were cultured in Eagle’s minimal essential medium (MEM; Calbiochem, San Diego, CA) supplemented with 5% fetal bovine serum (FBS), 1% penicillin/streptomycin, and 2 mM l-glutamine (Life Technologies, Inc. Rockville, MD) at 37°C in a humidified atmosphere with 5% CO_2_ in the air. To investigate the potential contribution of the NFκB transcription factor to COX-2 transcriptional induction by arsenite, we used COX-2-luciferase (COX-2-Luc) reporter containing full length (−1432/+59) or a mutation of the NFκB binding sites (−223/−214) of human COX-2 gene promoter linked to the luciferase (Subbaramaiah et al. 2001; Yan et al. 2000) and/or with IKKβ-KM as described previously (Ouyang et al. 2006). Wild-type and IKKβ knockout (IKKβ^−/−^) mouse embryonic fibroblasts (MEFs) were cultured in Dulbecco’s modified Eagle’s medium (DMEM) containing 10% FBS, 1% penicillin/streptomycin, and 2 mM l-glutamine.

### Construction of the siRNA vector

The specific small-interference RNA (siRNA)–targeted mouse COX-2 was designed using the siRNA converter of Ambion Inc. (2006a) according to the gene sequence in GenBank (mouse NM-011198, National Center for Biotechnology Information 2006b) and guidelines for siRNA (Ambion Inc. 2006b); the siRNA was synthesized by Invitrogen (Carlsbad, CA). The target sequence for mouse COX-2 was 5′-AGACAGATCATAAGCGAGGA-3′. The siRNA sequence was controlled via BLAST search (National Center for Biotechnology Information 2006a) and did not show any homology to other known genes. The siRNA was then inserted into pSuppressor vector and verified by DNA sequencing. The siRNA vector was designated as siCOX-2.

### Stable transfection

Cl41 cells were transfected with either siCOX-2 or small-interference–green fluorescent protein. IKKβ^−/−^ MEFs were transfected with COX-2 expression vector, which was a gift from K. Subbaramaiah (Weill Medical College of Cornell University, New York, NY). The transfection was performed by Lipofectamine 2000 reagent (Gibco BRL, Rockville, MD) according to the manufacturer’s instructions. Briefly, the cells were cultured in a 6-well plate to 85–90% confluence. Five micrograms of plasmid DNA was mixed with 10 μL Lipofectamine 2000 reagent and then used to transfect each well in the absence of serum. After 4–6 hr, the medium was replaced with 5% FBS MEM for Cl41 cells or 10% FBS DMEM for MEFs. Approximately 36–48 hr after the beginning of the transfection, the cells were cultured with medium containing 500 μg/mL G418 (Gibco BRL). After selection for 28–45 days with G418, the stable transfectants were identified by Western blot. Stable transfectants, Cl41-mock, Cl41-siCOX-2, IKKβ^−/−^(vector), and IKKβ^−/−^(COX-2) were established and cultured in G418-free medium for at least two passages before each experiment.

### COX-2 expression assay

We cultured 2 × 10^5^ Cl41 cells, IKKβ^−/−^ MEFs, and their transfectants in each well of 6-well plates to 70–80% confluence. After exposure to arsenite for indicated times, the cells were washed once with ice-cold phosphate-buffered saline (PBS) and then extracted with sodium dodecyl sulfate (SDS) sample buffer. The cell extracts (with GAPDH used as a control for protein loading) were separated on polyacrylamide-SDS gels, transferred, and probed with a rabbit-specific antibody against COX-2 (Cayman Chemical, Ann Arbor, MI). The protein band, specifically bound to the primary antibody, was detected using an anti-rabbit IgG-alkaline phosphatase-linked antibody and an enhanced chemifluorescence Western blotting system (Amersham Biosciences, Piscataway, NJ).

### Cell apoptosis analysis by flow cytometry

Cells (2 × 10^5^) were seeded into each well of 6-well plates and cultured to 70–80% confluence. After exposure to arsenite, the cells were harvested and fixed with 3 mL ice-cold 80% ethanol overnight. The fixed cells were washed twice with PBS and then suspended in propidium iodide (PI) staining solution (50 μg/mL PI, 10 mg/mL RNase A) (Sigma Chemical, St. Louis, MO) for at least 1 hr at 4°C. Cell apoptosis was determined by flow cytometry using the Epics XL FACS and EXPO 32 software (Beckman Coulter, Miami, FL) as described previously (Ouyang et al. 2006).

### TUNEL assay

We performed the TUNEL assay using an *in situ* cell death detection kit (Roche Applied Science, Indianapolis, IN) following the kit instructions. Briefly, the exposed cells were fixed by 4% polyparaformaldehyde solution in PBS for 24 hr at room temperature. After rinsing with PBS, the cells were resuspended in a solution with 0.1% Triton X-100 and 0.1% sodium citrate for 5 min to increase permeability of the cell membrane, and then incubated with 50 μL TUNEL reaction mixture containing terminal deoxynucleotidyl transferase (TdT) and fluorescein isothiocyanate-deoxyuridine triphosphate (FITC-dUTP) for 60 min at 37°C. After washing, the label incorporated at the damaged sites of the DNA was visualized by flow cytometry using the Epics XL FACS and EXPO 32 software.

## Results

### Arsenite exposure induced COX-2 expression in Cl41 cells through the IKKβ/NFκB pathway

**Figure 1:**
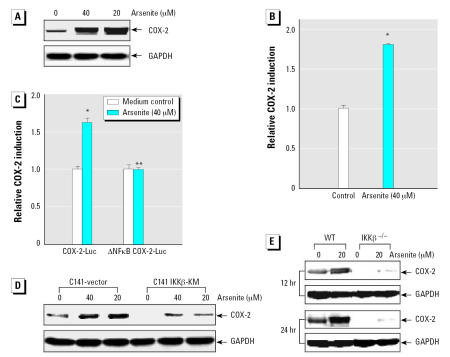
COX-2 expression induced by arsenite exposure through the IKKβ/NFκB pathway. (*A*) C141 cells were treated with various concentrations of arsenite as indicated for 12 hr, extracted, and then analyzed by Western blot with specific antibodies against COX-2 or GAPDH, as described in “Materials and Methods.” (*B, C*) Relative COX-2 induction (activity relative to control) in Cl41 cells treated with 40 μM arsenite for 12 hr (*B*) and stably transfected with COX-2-Luc reporter containing full length (−1432/+59) or mutation of the NFκB binding sites (−223/−214) of the human COX-2 gene promoter linked to luciferase (*C*); each bar indicates the mean ± SD of triplicate wells. (*D, E*) Cl41 cells transfected with control vector or IKKβ-KM (*D*) or wild-type MEFs and IKKβ^−/−^ MEFs (*E*) were exposed to arsenite for 12 or 24 hr and then subjected to Western blot assay. *Significant increase compared with medium control. **Significant decrease compared with intact COX-2 luciferase induction (*p* < 0.01).

Previous studies demonstrated that arsenite exerts its carcinogenic effect mainly by activating signal pathways and inducing gene expression involved in the regulation of cell proliferation and apoptosis (Huang et al. 2004; Pi et al. 2005; Rossman 2003; Yang and Frenkel 2002). COX-2, a key inducible enzyme in the biosynthesis of prostaglandins, has been related to inflammation, apoptosis, and carcinogenesis (Liu et al. 1998; Tsujii and DuBois 1995; Tsujii et al. 1997, 1998). To determine whether COX-2 is also involved in cell response to arsenite exposure, we examined COX-2 induction by arsenite in mouse epidermal Cl41 cells. As determined by Western blot analysis (Figure 1A), arsenite exposure caused a significant elevation of COX-2 protein level. Moreover, Cl41 cells exposed to arsenite for 12 hr showed a marked induction of COX-2 transcription in the gene reporter assay (Figure 1B).

The promoter region of the *COX-2* gene contains a canonical TATA box and multiple putative transcriptional regulatory elements, including NFκB, which has been indicated to be activated in Cl41 cells by arsenite exposure (Li et al. 2004). We investigated the potential contribution of the NFκB transcription factor to COX-2 transcriptional induction by arsenite using the COX-2-Luc reporter containing full length (−1432/+59) or mutant NFκB binding sites (−223/−214) of the *COX-2* gene promoter. As shown in Figure 1C, deletion of NFκB binding sites impaired arsenite-induced COX-2 transcriptional induction. Moreover, the stable transfectants of Cl41 cells harboring IKKβ-KM, a dominant mutant of IKKβ (Ouyang et al. 2006), and IKKβ^−/−^ MEFs were used to further confirm the requirement of the IKKβ/NFκB pathway for the induction of COX-2 by arsenite. Arsenite-induced COX-2 expression was dramatically inhibited in the IKKβ-KM-transfected Cl41 cells, as well as in IKKβ^−/−^ MEFs, when compared with control vector-transfected Cl41 cells or wild-type MEFs (Figure 1D, E). The basal level of COX-2 varied at different time points, which might be due to cell cycle progression (Figure 1D, E). Collectively, these results indicate that arsenite can induce COX-2 expression at both protein and transcription levels via an IKKβ/NFκB–dependent pathway, suggesting that COX-2 is involved in cell response to arsenite exposure.

### COX-2 induction through the IKKβ/NFκB pathway exerted an antiapoptotic effect on cells exposed to arsenite

**Figure 2:**
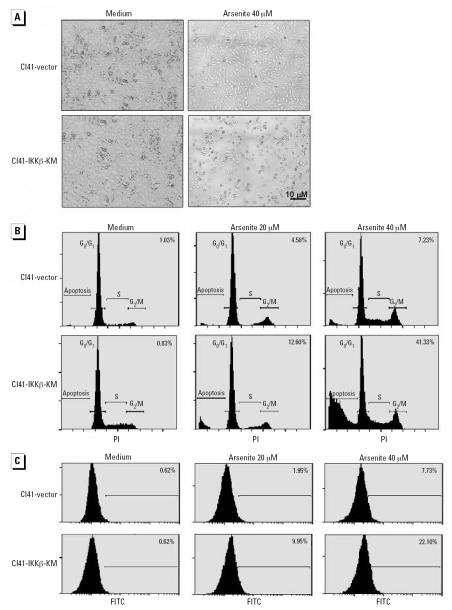
Transfection of IKKβ-KM increased the susceptibility of Cl41 cells to arsenite-induced cell apoptosis. C141-vector and Cl41-IKKβ-KM cells were treated with arsenite for 24 hr and photographed under microscopy (*A*). Cell apoptosis was analyzed using either PI staining (*B*) or the TUNEL assay (*C*) followed by flow cytometry analysis. (*B*) and (*C*) show representative results of three independent experiments; the numbers indicate the percentage of cells in sub-G_1_ phase (*B*) and the percentage of cells with positive TUNEL staining (*C*).

**Figure 3:**
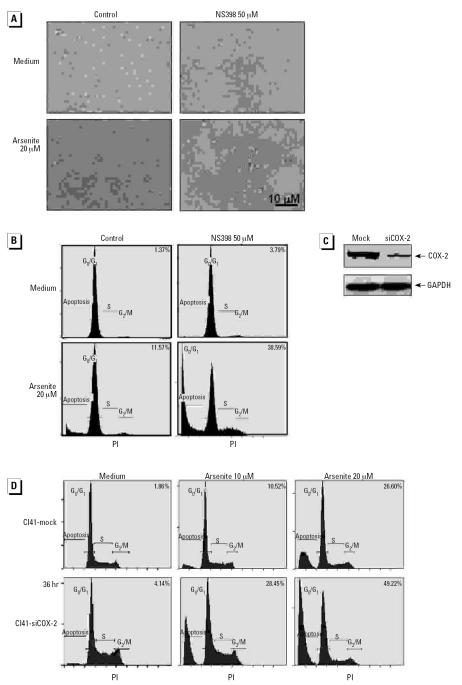
COX-2 induction is required for the protection of Cl41 cells from apoptosis after arsenite exposure. Cl41 cells were pretreated with 50 μM NS398 for 0.5 hr, treated with 20 μM arsenite for 48 hr, and photographed under microscopy (*A*); cell apoptosis was then analyzed by PI staining (*B*). (*C*) C141-mock vector and Cl41-siCOX-2 cells were exposed to 20 μM arsenite for 12 hr and then extracted with SDS-sample buffer; the cell extracts were analyzed by Western blot with antibodies against COX-2 or GAPDH. (*D*) C141-mock vector and Cl41-siCOX-2 cells were treated with arsenite for 36 hr, and cell apoptosis was analyzed using PI staining followed by flow cytometry analysis. Numbers in (*B*) and (*D*) indicate the percentage of cells in sub-G_1_ phase.

In view of the importance of COX-2 in the regulation of cell apoptotic response in some cells, we proposed that the induction of COX-2 may also be implicated in the regulation of cell apoptosis upon arsenite exposure. Based on the above results that the IKKβ/NFκB pathway was required for COX-2 induction in the cells exposed to arsenite, we examined the apoptosis of Cl41 cells transfected with IKKβ-KM after the exposure to arsenite. The results obtained from microscopic observation of cell morphology (Figure 2A), DNA content analysis by PI staining followed by flow cytometry analysis (Figure 2B), and DNA fragment detection by TUNEL assay followed by flow cytometry analysis (Figure 2C) showed that the transfection of IKKβ-KM made Cl41 cells much more sensitive to apoptotic induction by arsenite. To confirm the importance of COX-2 in the regulation of apoptotic response after arsenite exposure, we pretreated Cl41 cells with NS398, an inhibitor of COX-2, and found that it significantly sensitized the cells to arsenite-induced cell apoptosis (Figure 3A, B), suggesting that COX-2 may be the mediator responsible for the antiapoptotic effect. This notion was further confirmed by the finding that knockdown of endogenous COX-2 expression by its specific siRNA rendered Cl41 cells much more susceptible to cell apoptotic induction by arsenite (Figure 3C, D).

**Figure 4:**
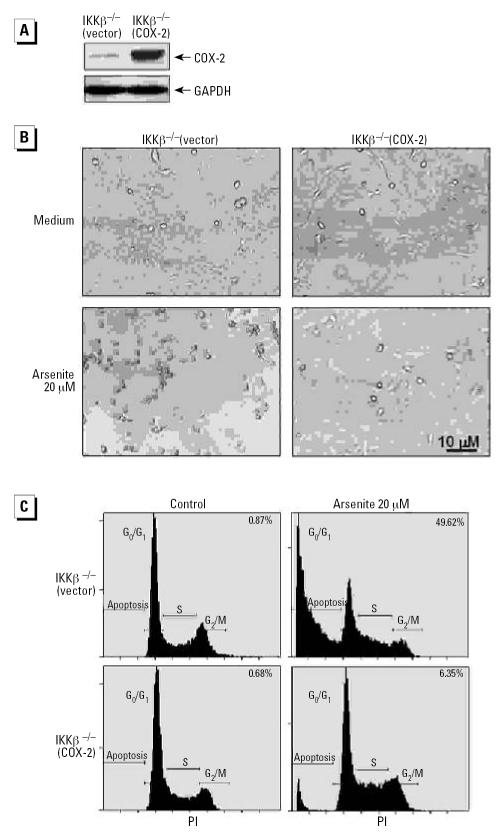
IKKβ^−/−^ MEFs overexpressing COX-2 acquired resistance to arsenite-induced cell apoptosis. (*A*) IKKβ^−/−^(vector) and IKKβ^−/−^(COX-2) cells were extracted with SDS-sample buffer, and the cell extracts were analyzed by Western blot with antibodies against COX-2 or GAPDH. (*B, C*) IKKβ^−/−^(vector) and IKKβ^−/−^(COX-2) were treated with 20 μM arsenite for 36 hr and photographed under microscopy (*B*); the cell apoptosis was analyzed using PI staining followed by flow cytometry analysis (*C*). Numbers in (*C*) indicate the percentage of cells in sub-G_1_ phase.

The role of COX-2 induction in protecting cells from apoptosis after arsenite exposure was further verified by the finding that over-expression of COX-2 in IKKβ^−/−^ MEFs made the cells much more resistant to arsenite-induced apoptosis (Figure 4). Collectively, these results demonstrate that COX-2 induction through the IKKβ/NFκB pathway can protect arsenite-exposed cells from apoptosis.

## Discussion

Arsenite is a well-documented skin carcinogen (Landolph 1994; Nriagu 2002); skin lesions, including skin cancers, are characteristic of exposure to arsenite in drinking water (Haque et al. 2003). Given the low genotoxic activity, arsenite is thought to exert its carcinogenic effect mainly through inducing activation of signal pathways, which thereby affects the expression of genes involved in regulating the machineries of the cell cycle and apoptosis (Huang et al. 2004; Pi et al. 2005; Rossman 2003; Yang and Frenkel 2002). In the present study, we have addressed the events involved in the regulation of apoptosis of cells exposed to arsenite, and demonstrated that induction of COX-2 expression through the IKKβ/NFκB pathway plays a role in antagonizing cell apoptosis caused by arsenite in mouse epidermal Cl41 cells.

The effect of arsenite on COX-2 expression depends on cell type and arsenite dosage. Arsenite stimulates COX-2 expression in endothelial cells through activating IKK/NFκB and extracellular signal–regulated kinases, respectively (Trouba and Germolec 2004; Tsai et al. 2002), whereas in a recent study, Ding et al. (2006) found that arsenite induces COX-2 expression in human bronchial epithelial Beas-2B cells through NFAT (nuclear factor of activated T cells) rather than NFκB and activator protein-1. Arsenite has been demonstrated to repress constitutive activation of NFκB and COX-2 expression in human acute myeloid leukemia (HL-60) cells (Han et al. 2005), and pretreatment of arsenite attenuates benzo[*a*]pyrene cytotoxicity in a human lung adenocarcinoma cells by decreasing cyclooxygenase-2 levels (Ho and Lee 2002). In the present study, we provide the first evidence that arsenite can induce COX-2 expression through the IKKβ/NFκB pathway in mouse epidermal Cl41 cells.

Although the detailed mechanisms underlying tumorigenesis remain largely undefined, it is generally accepted that apoptosis evasion is one of the hallmarks during cancer development (Hanahan and Weinberg 2000). Apoptosis plays a major role in developmental biology, cellular population dynamics, and disease states. Apoptosis typically occurs when cellular genetic damage exceeds the repair capacity. The suppression of apoptosis, in the face of significant genetic damage, could facilitate accumulation of aberrant cells and may be a critical step in the pathogenesis of malignancy (Abrams 2002; Johnstone et al. 2002; Zornig et al. 2001). As a sensor of cellular stress, p53 is a critical initiator of the apoptotic pathway (Lowe and Lin 2000). p53 protein accumulates in cells under stress, which thereby promotes apoptosis mainly by activating the expression of proapoptotic Bcl-2 family members (e.g., Bax, Bak, PUMA, Noxa) and repressing antiapoptotic Bcl-2 (B-cell leukemia) proteins (Bcl-2, Bcl-XL) and inhibitor of apoptosis protein (survivin) (Bartke et al. 2001; Hoffman et al. 2002; Ryan et al. 2001; Wu et al. 2001). The elimination of these damaged cells through apoptosis maintains genomic stability and prevents tumorigenesis. Because p53 mediates cell apoptosis and growth arrest, p53 mutation is responsible for > 50% of cancer development in humans. In the present study, we demonstrated that COX-2 plays an important role in antagonizing cell apoptosis induced by arsenite in mouse epidermal cells. Although a large body of evidence indicates the importance of COX-2 in the regulation of cell apoptosis, the mechanisms are not well-defined. Nonetheless, there is evidence supporting that COX-2 may interfere with p53-mediated cell apoptosis (Han et al. 2002) and regulate mitochondrial-triggered cell apoptosis (Sun et al. 2002). Although the exact mechanisms require further investigation, the antiapoptotic effect of COX-2 observed in the present study may provide more strategies with COX-2 as the target for skin cancer prevention and skin cancer therapy, especially in those countries with high arsenite contamination in drinking water.

It is notable that the contributions of the IKKs/NFκB pathway to carcinogen- induced skin cancer remain controversial. IKKα has been demonstrated to be an inhibitory factor for the proliferation of skin epidermis (Hu et al. 1999, 2001; Li et al. 1999) and over-expression of active p50 and p65 NFκB subunits in transgenic epithelium-produced hypoplasia and growth inhibition (Seitz et al. 1998). However, it has been reported that the deletion of IKKβ does not affect the proliferation of skin epidermis (Pasparakis et al. 2002); IκBα deficiency results in a sustained NFκB response and severe widespread dermatitis characterized by epidermal hyperplasia in mice (Klement et al. 1996). Budunova et al. (1999) demonstrated that epidermal inflammation and hyperplasia play a critical role in skin tumor promotion, and NFκB is one of the well-known mediators of these effects. Substances such as phorbol ester and okadaic acid, which are promoters of skin cancer, are also strong inducers of the NFκB response in keratinocytes (Budunova et al. 1999). In the present study, we demonstrated that the IKKβ/NFκB pathway is required for COX-2 induction by arsenite, suggesting that the IKKβ/NFκB pathway may contribute to arsenite-induced carcinogenesis by protecting cells from apoptosis through inducing COX-2 expression. Interestingly, we also found that apoptosis of IKKβ^−/−^ MEFs induced by arsenite is affected largely by cell density. High density of IKKβ^−/−^ MEFs shows much lower susceptibility to arsenite-induced apoptosis (Song et al. 2006). The mechanisms are now under investigation in our laboratory.

In summary, we have demonstrated that exposure of the cells to arsenite causes a significant COX-2 expression in an IKKβ/NFκB–dependent manner, which thereby plays an important role in antagonizing apoptosis induced by arsenite. These results suggest that arsenite, as a carcinogen, may facilitate skin cancer development by supporting the survival of the cells with genetic alterations, which is usually eliminated by apoptosis. Thus, inhibition of COX-2 may be a promising approach for skin cancer prevention in those countries with severe arsenite pollution in drinking water.
